# The gut–brain axis in Alzheimer’s disease: how gut microbiota modulate microglial function

**DOI:** 10.3389/fragi.2025.1704047

**Published:** 2025-11-21

**Authors:** Huan Wang, Feifan Yang, Zhejianyi Gao, Zedong Cheng, Xicai Liang

**Affiliations:** 1 College of Acupuncture-Moxibustion and Tuina, Liaoning University of Traditional Chinese Medicine, Shenyang, China; 2 Graduate School of Engineering, Tokyo University of Agriculture and Technology, Tokyo, Japan; 3 College of Laboratory Animal Medicine, Liaoning University of Traditional Chinese Medicine, Shenyang, China

**Keywords:** Alzheimer’s disease, intestinal flora, microglia, neuroinflammation, treatment strategy

## Abstract

Alzheimer’s disease (AD) is a complex neurodegenerative disorder that can be caused by multiple factors, such as abnormal amyloid-beta (Aβ) deposition, pathological changes in Tau protein, lipid metabolism disorders, and oxidative stress. Recent studies have revealed the potential link between gut microbiota and AD, particularly the impact of gut microbiota and its derivatives on microglia. As immune cells in the central nervous system (CNS), microglia are involved in neuroinflammation and the regulation of cognitive function. Research indicates that the dysregulation of gut microbiota may affect the phenotype and function of microglia through various mechanisms, including direct metabolite action and indirect immune and neurotransmitter regulation. This article reviews the direct and indirect effects of gut microbiota and its derivatives on microglia, explores their role in the pathogenesis of AD, and discusses therapeutic strategies based on gut microbiota, such as dietary regulation, probiotics, fecal microbiota transplantation, and traditional Chinese medicine. Although existing studies have shown the potential of these interventions, further research is needed to completely understand their application in the treatment of AD.

## 1 Introduction

Alzheimer’s disease (AD) is a neurodegenerative disorder characterized by insidious onset and progressive decline in cognitive abilities, including learning and memory functions. The disease primarily manifests as a gradual impairment of cognitive function and is often accompanied by a range of psychiatric and behavioral symptoms (Scheltens et al., 2021). As global populations age, the worldwide prevalence of dementia is projected to increase from 55 million in 2019 to 139 million by 2050 (Gaugler et al., 2019). Alzheimer’s disease and other dementias are estimated to cost the global economy $1,451.3 billion between 2020 and 2050, equivalent to 0.421% of global GDP per year (Chen et al., 2024). As a consequence, AD is emerging as one of the most formidable public health challenges of the 21st century. The etiology of AD is complex and diverse, and the precise mechanisms underlying its onset are not yet completely understood (Zhang et al., 2024; Wang et al., 2025). For decades, research into AD has been dominated by the amyloid cascade hypothesis (Hunter et al., 2025). However, amyloid-beta (Aβ) clearance alone slows progression by only 35% (Rafii and Aisen, 2025). This compels increasing attention to peripheral factors in AD pathophysiology (Dammer et al., 2025), redirecting the field from a brain-centric, amyloid-focused model toward a systemic perspective that emphasizes peripheral–central interactions. It is now increasingly recognized that chronic, low-grade systemic inflammation, a condition often termed “inflammaging,” acts as a critical driver of neuroinflammation and accelerates neurodegenerative processes (Shi and Yong, 2025). Within this framework, the gastrointestinal tract, which harbors the body’s largest immune cell population and the vast metabolic capacity of the gut microbiome, emerges as a pivotal hub for originating peripheral signals that shape brain health and disease.

The human gastrointestinal tract harbors a vast and diverse population of microorganisms, collectively known as the gut microbiota. It has been estimated that the number of bacteria in the gut reaches up to 100 trillion, and their collective genetic content is approximately 450 times larger than the human genome, earning the designation of “the second human genome” (Sender et al., 2016). Recent clinical investigations have demonstrated that alterations in the gut microbiota constitute an early and persistent pathological event in the course of Alzheimer’s disease (Jia et al., 2025). Moreover, clinical studies have revealed significant alterations in gut microbial composition and function in patients with Alzheimer’s disease compared with those in cognitively healthy individuals, characterized by an increased abundance of pro-inflammatory taxa (e.g., *Bacteroides* and *Fusobacterium*) and a reduction in beneficial commensals (e.g., *Clostridium* and Turicibacter) (Kang et al., 2025). Several human investigations further support the biological relevance of this dysbiosis: gut microbiota signatures have been proposed as early biomarkers in preclinical AD (Ferreiro et al., 2023). This complex bidirectional communication network between the gut and the brain is referred to as the microbiota–gut–brain axis (MGBA) (Yoo and Mazmanian, 2017). Notably, the gut microbiota, through the MGBA, is crucial in modulating neuroinflammation (Lei et al., 2025). Dysbiosis disrupts gut barrier integrity, promotes systemic inflammation, and exacerbates neuroinflammatory responses, thereby accelerating AD progression. Recent advances reveal that gut microbiota-derived metabolites [e.g., short-chain fatty acids (SCFAs) and lipopolysaccharides (LPSs)] influence microglial activation and Aβ aggregation (Qian et al., 2023; Ling et al., 2021).

Central to this gut–brain inflammatory crosstalk are microglia, the resident immune cells of the central nervous system (CNS) (Keane et al., 2025), as the first line of defense against pathogens, regulating neuroinflammation and influencing CNS functions (Lopez et al., 2019; Lyu et al., 2021). Genome-wide association studies (GWASs) have robustly identified numerous AD risk loci, such as TREM2 and CD33, which are predominantly expressed in microglia, positioning these cells as critical mediators of disease susceptibility (Wightman et al., 2021; Guerreiro et al., 2013; Jonsson et al., 2013). Crucially, microglia are highly dynamic sensors of their environment; their transition from a homeostatic, surveillance state to a reactive, disease-associated phenotype is tightly regulated by molecular cues from both central and peripheral origins (Zhang et al., 2025). Mounting evidence points to the gut microbiota and its derivatives as a major source of these peripheral cues. Signals such as microbial-associated molecular patterns (MAMPs) including LPS and neuroactive metabolites can cross a compromised intestinal barrier (IB) and blood–brain barrier (BBB), directly engaging with microglial receptors (Eid et al., 2025). This interaction can “prime” microglia, lowering their threshold for activation and leading to an exaggerated neuroinflammatory response to endogenous stimuli such as Aβ aggregates, thereby creating a vicious cycle that accelerates neurodegeneration (Brown and Heneka, 2024).

Despite this burgeoning understanding, critical knowledge gaps persist. For instance, is gut dysbiosis a causative initiator of AD pathology, or merely a consequence of the disease process? Furthermore, the molecular specificity by which distinct microbiota-derived molecules—from beneficial short-chain fatty acids to detrimental factors, including LPS—dictate the functional polarization of microglia remains to be fully elucidated. Therefore, this review aims to move beyond a general overview and systematically dissect these complex interactions. We summarize the current research on the direct and indirect mechanisms by which gut microbes and their metabolites affect the phenotype and function of microglial cells in AD. By exploring this intricate communication, we aim to provide a comprehensive perspective on the pathogenesis of AD and offer insights into novel therapeutic strategies targeting the microbiota–gut–brain axis.

## 2 Direct effects of gut microbiota and its derivatives on microglia

**FIGURE 1:**
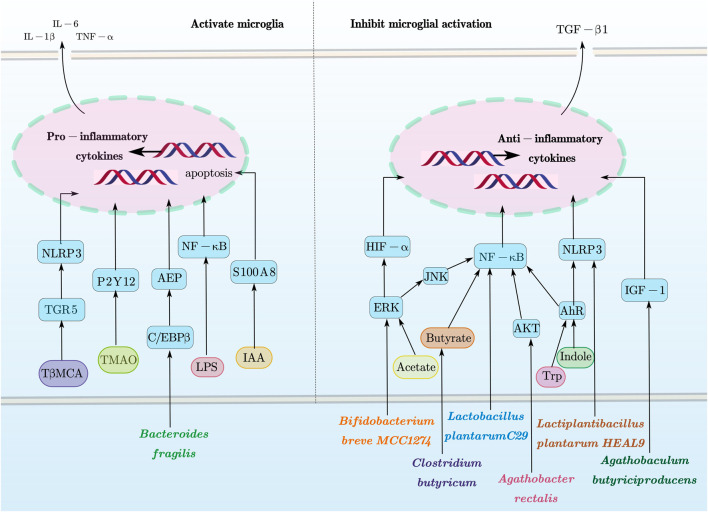
Direct effects of gut microbiota and its derivatives on microglia. This figure provides a schematic overview of how gut microbiota and their metabolites modulate microglial functions via these signaling pathways as discussed in this review. In particular, microglial activation and apoptosis are closely associated with pathways such as ERK, JNK, AKT, AhR, NLRP3, IGF-1, HIF-α, and NF-κB. Conversely, microglial inhibition is mediated through pathways including TGR5, P2Y12, AEP, S100A8, and C/EBPβ, along with the context-dependent NLRP3 and NF-κB pathways. This figure was created with AxGlyph. ERK, extracellular signal-regulated kinase; JNK, c-Jun N-terminal kinase; AKT, protein kinase B; AhR, using hydrocarbon receptor; NLRP3, NOD-, LRR-, and pyrin domain-containing protein 3 receptor; IGF-1, insulin-like growth factor 1; HIF-α, hypoxia-inducible factor-alpha; NF-κB, nuclear factor kappa-light-chain-enhancer of activated B cells; TGR5, Takeda G protein-coupled receptor 5; P2Y12, purinergic receptor P2Y, G-protein coupled 12; AEP, asparagine endopeptidase; S100A8, S100 calcium-binding protein A8; C/EBPβ, CCAAT/enhancer-binding protein beta.

The IB is composed of the epithelial layer covering the gut, along with associated elements such as the mucus layer, tight junctions, and immune cells, which coordinate selective permeability to gut contents and protect against pathogens and toxins (Pellegrini et al., 2018). The BBB is formed by specialized endothelial cells in the microvasculature, regulating the exchange of molecules and nutrients between the blood and brain tissue (Obermeier et al., 2013). Dysbiosis of the gut microbiota can impair the integrity of the IB, potentially triggering or exacerbating inflammation at the IB; this may allow pathogenic microorganisms to cross the BBB unimpeded (Kurita et al., 2020), thereby affecting the maturation, morphology, and function of microglia (Liu et al., 2023), ultimately leading to neuroinflammation, neurodegeneration, and age-related brain pathology (Mou et al., 2022). The direct effects of gut microbiota and its derivatives on microglia are summarized in Figure 1.

### 2.1 Direct effects of gut microbiota on microglia

#### 2.1.1 Agathobaculum butyriciproducens

Agathobaculum butyriciproducens is an anaerobic, Gram-positive, non-spore-forming, non-motile, catalase- and oxidase-negative, flagellum-lacking, short rod-shaped bacterium (Ahn et al., 2016). It produces butyrate and is a part of the gut microbiota, playing a crucial role in gut health and energy metabolism. A study found that butyrate is more abundant in young mice compared to aged mice (Chilton et al., 2024). In APP/PS1 transgenic mice, butyrate-producing bacteria, such as Eubacteria, Roseburia, and Clostridia, are reduced compared to normal mice (Abraham et al., 2019). Further research demonstrated that Agathobaculum butyriciproducens can inhibit the activation of microglia, improving cognitive function in LPS-induced cognitive impairment mouse models and APP/PS1 transgenic mouse models (Go et al., 2021). *Clostridium* butyricum (CB), a strain of Agathobaculum butyriciproducens, has been shown to inhibit the activation of microglia and reduce the levels of pro-inflammatory cytokines in APP/PS1 transgenic mice after a 4-week intervention. *In vitro* experiments have further demonstrated that butyrate produced by CB can inhibit the activation of BV2 microglial cells by suppressing the phosphorylation of nuclear factor kappa-light-chain-enhancer of activated B cells p65 (NF-κB p65), reducing the levels of integrin alpha-M (CD11b) and cyclooxygenase-2 (COX-2), thereby alleviating microglia-mediated neuroinflammation (Sun J. et al., 2020).

#### 2.1.2 Bacteroides fragilis

*Bacteroides fragilis* is a Gram-negative, obligate anaerobic bacterium, constituting approximately 30% of the gut microbiota in the gastrointestinal tract (Fathi and Wu, 2016). Studies have shown that treatment with *Bacteroides fragilis* in APP/PS1 mice increases Aβ plaques in female mice and downregulates the expression of genes related to microglial phagocytosis and protein degradation. Further experiments involving the injection of *Bacteroides fragilis* into aged wild-type (WT) male and female mice revealed that it inhibits microglial uptake of Aβ injected into the hippocampus. Moreover, treatment with metronidazole to deplete *Bacteroides fragilis* in aged 5xFAD mice leads to increased amyloid protein accumulation in the hippocampus and activation of microglial pathways associated with phagocytosis, cytokine signaling, and lysosomal degradation. These findings suggest that *Bacteroides fragilis* inhibits microglial phagocytic function, leading to impaired Aβ clearance and the accumulation of amyloid plaques, thereby contributing to the pathogenesis of AD (Wasén et al., 2024). Further studies have demonstrated through *in vivo* and *in vitro* experiments that *Bacteroides fragilis* and its metabolites, 12-hydroxyheptadecatrienoic acid (12-HHTrE) and prostaglandin E2 (PGE2), can activate microglial cells (Xia et al., 2023), increasing the expression of pro-inflammatory cytokines such as interleukin-1 beta (IL-1β) and interleukin-6 (IL-6), thereby exacerbating neuroinflammation, leading to Aβ plaque deposition and tau protein phosphorylation, and ultimately affecting cognitive function and memory. Additional research indicates that *Bacteroides* strains are increased in the gut microbiota of AD patients. These strains mediate the metabolism of pro-inflammatory polyunsaturated fatty acids (PUFAs), which may be directly involved in the metabolism of arachidonic acid (AA), thereby affecting the production of PGE2 and regulating microglial activation (Chen C. et al., 2022).

#### 2.1.3 Bifidobacteria

Bifidobacteria are Gram-positive, anaerobic or microaerophilic bacteria belonging to the phylum Actinobacteria. A study demonstrated that a 6-month treatment with bifidobacteria reduces Aβ deposition in the brains of APP/PS1 mice, inhibits the activation of microglia, and decreases the release of inflammatory factors such as IL-1β, IL-6, tumor necrosis factor-alpha (TNF-α), interleukin-4 (IL-4), and interferon-gamma (IFN-γ), thereby attenuating neuroinflammation (Wu et al., 2020). Bifidobacterium breve MCC1274 (B. breve MCC1274) reduces the number and activation of microglia in the hippocampus of mice and may promote the transition of microglia from a pro-inflammatory phenotype to an anti-inflammatory phenotype, thereby exerting neuroprotective effects (Abdelhamid et al., 2022a). Additionally, multiple studies have shown that bifidobacteria can inhibit the polarization of microglia, alleviate neuroinflammation, and improve cognitive deficits (Zhu et al., 2023; Abdelhamid et al., 2022b). Mariano et al. examined plasma homocysteine levels, serum folate and vitamin B12 concentrations, plasma pyridoxal phosphate levels, C-reactive protein, and antibodies of immunoglobulin G (IgG) and immunoglobulin A (IgA) against *Helicobacter pylori* (HP) in 30 AD patients and found an association between HP infection and AD (Malaguarnera et al., 2004). Urease (HPU) is an enzyme produced by HP. A previous study demonstrated that HPU-treated BV2 microglia produce reactive oxygen species (ROS) and cytokines IL-1β and TNF-α while exhibiting reduced cell viability. After intraperitoneal injection of HPU in rats, excessive expression of the microglial activation marker Iba1 was observed, but HPU was not detected in brain homogenates. It was concluded that HP infection might affect AD by inhibiting microglial activation through its urease production (Uberti et al., 2022).

#### 2.1.4 Lactobacillus

Lactiplantibacillus plantarum HEAL9 is a specific strain of Lactiplantibacillus plantarum, a Gram-positive rod-shaped bacterium belonging to the family Lactobacillaceae. A previous study showed that it can suppress microglial activation by inhibiting the NLRP3 inflammasome signaling pathway, thereby reducing neuroinflammation and improving cognitive function (Di Salvo et al., 2024). Researchers fed 5xFAD transgenic mice with *Lactobacillus plantarum* C29-fermented defatted soybean (FDS, DW2009) and *Lactobacillus plantarum* C29. The results indicated that oral administration of FDS or C29 increases cognitive function in mice, significantly suppresses amyloid-β, β/γ-secretases, caspase-3 expression, and NF-κB activation, activates microglia and apoptotic neuron cell populations, and increases brain-derived neurotrophic factor (BDNF) expression in the brain. Furthermore, treatment with FDS or C29 reduced lipopolysaccharide levels in the blood and feces, suppressed the abundance of Enterobacteriaceae, and increased the populations of lactobacilli and bifidobacteria (Lee et al., 2018).

#### 2.1.5 Agathobacter rectalis

Agathobacter rectalis is a bacterium belonging to the phylum Firmicutes and is a common member of the human gut microbiota. It belongs to the genus Agathobacter within the family Lachnospiraceae. A study found that the abundance of Agathobacter is significantly lower in AD patients and is negatively correlated with cognitive impairment. Subsequent animal experiments demonstrated that Agathobacter rectalis and its metabolite butyrate effectively inhibited the activation of microglia in APP/PS1 mice by modulating the AKT/NF-κB pathway, reducing the production of pro-inflammatory cytokines, and thereby alleviating neuroinflammation (Lv et al., 2024). The direct effects of gut microbiota on microglia are summarized in Table 1.

**TABLE 1:** Direct effects of gut microbiota on microglia.

Designation	Category	Mechanism	Impact on microglia	Impact on AD	References
*Agathobaculum butyriciproducens*	APP/PS1 mice	Activate the IGF-1 signaling pathway and decrease the gene expression levels of IL-1β and C1QB	Inhibit microglial activation	Alleviate	Go et al. (2021)
*Clostridium* *butyricum*	APP/PS1 mice	Decrease the expression of Iba1 and reduce the levels of TNF-α and IL-1β	Inhibit microglial activation	Alleviate	Sun J. et al. (2020)
*Bacteroides fragilis*	APP/PS1 mice	Inhibit the gene expressions of Trem2 and cathepsin L in microglia	Inhibit microglial phagocytic function	Aggravate	Wasén et al. (2024)
*Bacteroides fragilis*	Neuronal C/EBPβ transgenic mice	Activate the gene expression of C/EBPβ in microglia	Activate microglia	Aggravate	Xia et al. (2023)
Bifidobacteria	APP/PS1 mice	Decrease the expression of Iba1 and inhibit the release of IL-1β, TNF-α, IL-4, IL-6, and IFN-γ	Inhibit microglial activation	Alleviate	Wu et al. (2020)
*Bifidobacterium breve* MCC1274	APP^NL-G-F^ mice	Decrease the expression of Iba1, inhibit the expressions of IL-1β and IL-6, and promote the expression of TGF-β1	Reduce microglial activation levels, promoting the shift from a pro-inflammatory phenotype to an anti-inflammatory phenotype	Alleviate	Abdelhamid et al. (2022a)
*Helicobacter pylori* urease	BV2 microglia	Increase pro-inflammatory cytokines IL-1β and TNF-α and significantly decrease BV-2 cell viability	Activate microglia	​	Uberti et al. (2022)
*Lactiplantibacillus plantarum* HEAL9	SAMP8 mice	Inhibit the NLRP3 inflammasome signaling pathway	Inhibit microglial activation	Alleviate	Di Salvo et al. (2024)
*Lactobacillus* *plantarum* C29	5xFAD transgenic mice	Increase BDNF expression and CREB phosphorylation and inhibit NF-κB activation	Inhibit microglial activation	Alleviate	Lee et al. (2018)
*Agathobacter rectalis*	APP/PS1 mice	Regulate the Akt/NF-κB pathway	Inhibit microglial activation	Alleviate	Lv et al. (2024)

### 2.2 Direct effects of gut microbiota-derived metabolites on microglia

Gut microbiota-derived metabolites are categorized into three groups: (1) diet-derived products made directly by microbes (e.g., SCFAs); (2) host-synthesized metabolites structurally remodeled by microbes [e.g., secondary bile acids (BAs)]; and (3) *de novo* microbial products, including those first formed in the host and subsequently microbially modified. Notably, the gut microbiota shapes the production of lipid mediators—including PUFA derivatives, prostaglandins, and bile acids—that collectively modulate microglial inflammatory responses. This regulatory axis is exemplified by the capacity of *Bacteroides* strains to bias arachidonic acid metabolism toward the production of pro-inflammatory PGE_2_, directly activating microglia. In parallel, the microbiota-generated secondary bile acid TβMCA induces a pro-inflammatory M1-like microglial state, contributing to age-related behavioral deficits. Despite acting through distinct cell-surface receptors, the downstream signaling of these lipid mediators converges upon shared intracellular pathways, such as the NF-κB/MAPK cascades and the NLRP3 inflammasome. This mechanistic convergence establishes these lipid mediators as a critical communication channel through which the gut microbiome governs essential microglial functions, including inflammatory polarization, phagocytosis, and synaptic pruning.

#### 2.2.1 Short-chain fatty acids

SCFAs are major metabolites produced by the gut microbiota, generated by microbes such as Bacteroidetes and Firmicutes via fermentation of dietary fibers and resistant starches in the cecum and proximal colon. Acetate, propionate, and butyrate are the most abundant SCFAs, accounting for approximately 95% of total SCFAs in the human body. The effects of SCFAs on the pathogenesis of AD primarily involve epigenetic regulation, modulation of neuroinflammation, maintenance of the blood–brain barrier, regulation of brain metabolism, and interference with amyloid protein formation (Chen H. et al., 2022). A study found that SCFAs can attenuate the inflammatory response of microglia by decreasing the secretion of cytotoxins and pro-inflammatory cytokines, such as IL-1β, TNF-α, and monocyte chemoattractant protein-1 (MCP-1). Additionally, SCFAs can inhibit the phagocytic activity of microglia and reduce their capacity to produce ROS (Wenzel et al., 2020). An *in vivo* experiment showed that acetate can directly affect the maturation and homeostatic metabolic state of microglia, impacting the pathological progression of Alzheimer’s disease by inhibiting the phagocytosis of β-amyloid by microglia (Erny et al., 2021). An *in vitro* study demonstrated that acetate exerts anti-neuroinflammatory effects by upregulating G-protein-coupled receptor 41 (GPR41) and inhibiting the ERK/JNK/NF-κB signaling pathway, thereby suppressing the microglia activation (Liu et al., 2020). Butyrate, a butyrate salt derived from SCFAs, reduces the secretion of pro-inflammatory cytokines by inhibiting histone deacetylase (HDAC), thereby suppressing the overactivation of microglia and the accumulation of Aβ and improving synaptic plasticity (Jiang et al., 2021). Propionate, produced by gut bacteria through fermentation of dietary fibers (such as β-glucan), can regulate appetite and control blood glucose levels. It also plays roles in modulating immune cells, controlling intestinal inflammation, and maintaining the intestinal barrier. Although previous studies on the effects of propionate on microglia are limited, recent research has indicated that while propionate reduces microglial activation, it also impairs their phagocytic capacity, demonstrating a complex dual role in modulating microglial function (Gold et al., 2024).

#### 2.2.2 Tryptophan and indole derivatives

Tryptophan (Trp) is one of the essential amino acids metabolized by the gut microbiota and is obtained through the diet. It is considered a key player in host–gut microbiota communication (Khoshnevisan et al., 2022). Most dietary L-tryptophan released in the gut is transported into the circulatory system via epithelial cells, while approximately 10%–20% of L-tryptophan is metabolized by intestinal epithelial cells and the gut microbiota within the intestinal lumen (Dong and Perdew, 2020). Gut microbiota-mediated tryptophan metabolism involves several pathways, including the aryl hydrocarbon receptor (AhR) ligand pathway, the indole pathway, the kynurenine (Kyn) pathway, and the 5-hydroxytryptamine (5-HT) pathway (Giil et al., 2017; Ma et al., 2020; Gao et al., 2018; Agus et al., 2018; Sun M. et al., 2020). AhR is a ligand-activated transcription factor that can be activated by tryptophan and its derivatives (including indole, indole-3-propionic acid, and indole-3-acetic acid) (Borucki et al., 2018; Rothhammer et al., 2016). Indole activates the AhR signaling pathway, and upon AhR activation, the formation of the NLRP3 inflammasome is inhibited, leading to a reduction in the production of pro-inflammatory cytokines, including TNF-α, IL-6, IL-1β, and interleukin-18 (IL-18); decreased microglial hyperactivation; alleviated neuroinflammation; and improved cognitive and behavioral functions (Sun et al., 2022). Furthermore, studies have shown that Trp deficiency alters Trp-metabolizing bacteria in APP/PS1 mice, while a high-Trp diet significantly alleviates cognitive impairment and Aβ deposition. This effect is mediated through the regulation of the AhR/NF-κB signaling pathway, inhibition of microglial activation, and reduction in CD11b, COX-2, IL-1β, and IL-6 levels (Pan et al., 2024).

#### 2.2.3 Bile acids

BAs can be classified into primary and secondary bile acids. Primary bile acids are synthesized from cholesterol in the liver, transported through the biliary system, and released into the intestine. Primary bile acids can be further metabolized by the gut microbiota to produce secondary bile acids, which regulate intestinal mucosal immune homeostasis and inflammatory responses through interactions with their receptors and signaling pathways. A study suggested that bile acid metabolism disorders may play a critical role in the development of Alzheimer’s disease and hepatic encephalopathy (Jia et al., 2020). A clinical study involving 1,464 participants revealed that patients with AD had lower levels of primary bile acids [such as cholic acid (CA)] and higher levels of secondary bile acids [such as deoxycholic acid (DCA)] and conjugated bile acids [such as glycocholic acid (GCA) and taurocholic acid (TCA)] in their serum than those of cognitively normal elderly individuals (MahmoudianDehkordi et al., 2019). In an animal study, researchers first analyzed the bile acid profiles in the cerebral cortex, hippocampus, and hypothalamus of naturally aged mice and then identified a characteristic bile acid associated with aging—tauro-β-muricholic acid (TβMCA). It was found to increase the expression levels of inducible nitric oxide synthase (iNOS), serum amyloid A1 (Saa1), IL-18, intercellular cell adhesion molecule-1 (ICAM-1), TNF-α, and IL-6, indicating that microglia exhibited pro-inflammatory M1 activation, which induced neuroinflammation and behavioral impairments in mice (Ma et al., 2024).

#### 2.2.4 Trimethylamine N-oxide

Trimethylamine N-oxide (TMAO) is produced by gut microbiota through the metabolism of trimethylamine-containing nutrients such as choline, carnitine, and betaine into trimethylamine (TMA), which is subsequently oxidized in the liver by flavin-containing monooxygenases (FMOs). Studies on the impact of TMAO on AD have yielded conflicting results. Some studies have found that TMAO levels are elevated in the cerebrospinal fluid (CSF) of AD patients and are positively correlated with increased CSF biomarkers. However, a study where 5xFAD mice were supplemented with TMAO for 12 weeks revealed that it did not alter astrocyte and microglial responses or cortical synaptic protein expression. Instead, TMAO influenced AD pathology by reducing neurite density (Zarbock et al., 2022). More studies suggest that TMAO can cross the blood–brain barrier, triggering neurodegeneration by activating astrocytes and enhancing the release of inflammatory mediators (Brunt et al., 2021; Praveenraj et al., 2022). A minority of studies have reported that TMAO can downregulate the expression of P2Y12 receptors in microglia, increase inflammation in the paraventricular nucleus (PVN), and exacerbate sympathetic nervous system excitability, thereby exerting negative effects on the nervous system (Wang et al., 2024).

#### 2.2.5 Isoamylamine

Isoamylamine (IAA) is a biogenic amine produced by the dehydrogenation of isoamyl alcohol (IAC), which is generated by the fermentation of certain dietary components (such as amino acids) by gut microbiota. IAA is a neuroactive compound that can cross the blood–brain barrier and influence the central nervous system. IAA is associated with age-related cognitive decline, and it can induce microglial cell apoptosis, exerting its effects through the activation of the S100 calcium-binding protein A8 (S100A8) signaling pathway (Teng et al., 2022).

#### 2.2.6 Lipopolysaccharide

LPS is not a direct derivative of the gut microbiota but a unique component of the cell wall of Gram-negative bacteria. When Gram-negative bacteria in the intestinal microbiota undergo cell death and lysis, LPS is released into the gut environment, subsequently entering the bloodstream. Multiple lines of evidence suggest that aging combined with recurrent chronic infections can lead to exposure to exotoxins such as LPS and bacterial amyloid proteins, which can alter the activation of microglia, exacerbate inflammatory responses, and lead to Aβ fibrillation in the brain, thereby contributing to the pathogenesis of AD (Kesika et al., 2021). In particular, lipopolysaccharide secreted by *Bacteroides fragilis* (BF-LPS) can activate the inflammatory transcription factor NF-κB, thereby promoting the occurrence of inflammatory neurodegenerative diseases (Lukiw, 2016). Additionally, it can upregulate pro-inflammatory microRNAs (such as miRNA-34a and miRNA-146a), which can downregulate the expression of triggering receptors expressed on myeloid cells 2 (TREM2), thereby exacerbating neuroinflammation and Alzheimer’s disease pathology (Zhao and Lukiw, 2018). The direct effects of gut microbiota derivatives on microglia are summarized in Table 2.

**TABLE 2:** Direct effects of gut microbiota derivatives on microglia.

Designation	Category	Mechanism	Impact on microglia	Impact on AD	References
SCFAs—Acetate	5xFAD transgenic mice	Promote mitochondrial function to increase the energy supply of microglia and enhance their phagocytic capacity	Enhance the phagocytic capacity of microglia	Alleviate	Erny et al. (2021)
SCFAs—Acetate	BV2 microglia	Inhibit the expressions of CD11b, COX-2, and GPR41 in microglia and downregulate the phosphorylation levels of ERK, JNK, and NF-κB	Inhibit the activation of microglia	​	Liu et al. (2020)
SCFAs—Propionate	Immortalized murine microglia	Decrease the expressions of Cd36 and Msr1, increase the expression of Lpl, and impair the phagocytic ability of microglia	Decrease the activation of microglia and impair their phagocytic ability, with complex dual effects	​	Gold et al. (2024)
SCFAs—Butyrate	5xFAD transgenic mice	Reduce Iba-1 expression in microglia, decrease the production of TNF-α, IL-6, and IL-1β, and inhibit the NF-κB signaling pathway	Inhibit the activation of microglia	Alleviate	Jiang et al. (2021)
SCFAs—Butyrate	BV2 microglia	Inhibit the phosphorylation of NF-κB p65 in microglia and reduce the levels of CD11b and COX-2	Inhibit the activation of microglia	​	Sun M. et al. (2020)
Indole	APP/PS1 mice	Upregulate AhR expression and inhibit the formation of the NLRP3 inflammasome	Inhibit the activation of microglia	Alleviate	Khoshnevisan et al. (2022)
Trp	Male APP/PS1 mice	Regulate the AhR/NF-κB pathway to reduce CD11b expression	Inhibit the activation of microglia	Alleviate	Pan et al. (2024)
TβMCA	C57BL/6 J mice	Increase IBA1 expression and upregulate NOS, Saa1, IL-18, IL-6, ICAM1, and TNF-α expressions	Activate microglia	Aggravate	Ma et al. (2024)
TMAO	D-galactose-induced aging rats	Downregulate the expression of the P2Y12 receptor in microglia	Activate microglia	Aggravate	Wang et al. (2024)
IAA	BV2 microglia	Increase the expression levels of S100A8 and cleaved caspase-3	Induce apoptosis in microglia	​	Teng et al. (2022)
LPS	Human neuronal–glial cells and HNG cells	Activate the NF-κB signaling pathway, upregulate pro-inflammatory microRNAs (such as miRNA-34a and miRNA-146a), and downregulate TREM2 expression	Activate microglia	​	Zhao and Lukiw (2018)

## 3 Indirect effects of gut microbiota and its derivatives on microglia

### 3.1 Gut microbiota and its derivatives—immune system—microglia

**FIGURE 2:**
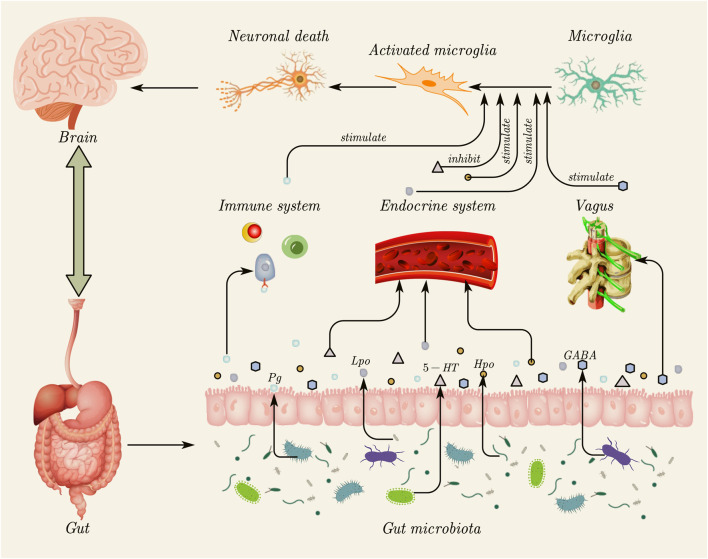
Indirect effects of gut microbiota and its derivatives on microglia. Gut microbiota can influence microglial activation through three primary indirect routes. (1) Immune system pathway: gut microbiota and their derivatives (e.g., Pg) can disrupt the intestinal barrier, triggering peripheral immune responses and promoting the release of pro-inflammatory cytokines (e.g., IFN-γ), which, in turn, stimulate microglia. (2) Endocrine system pathway: metabolites produced by gut microbiota, such as neurotransmitters (e.g., 5-HT) and other derivatives (Lpo and Hpo), enter the systemic circulation. These molecules can cross the blood–brain barrier to directly stimulate or inhibit microglial activity. (3) Vagus nerve pathway: intestinal microbiota derivatives (e.g., GABA) can be transmitted to the brain through the vagus nerve, indirectly stimulating microglia. The subsequent activation of microglia can contribute to neuronal death, highlighting the critical role of the gut–brain axis in regulating neuroinflammation. This figure was created using AxGlyph. Pg, Porphyromonas gingivalis; IFN-γ, interferon-γ; 5-HT, 5-hydroxytryptamine; Lpo, *Lactobacillus pentosus* OMVs; Hpo, *Helicobacter pylori* OMVs; GABA, gamma-aminobutyric acid.

Approximately 70%–80% of immune cells in the human body are located in the gastrointestinal tract, facilitating direct interactions between the gut and immune cells (Vighi et al., 2008). Disruption of the IB function increases the permeability of gut microbes, microbial derivatives (such as metabolites and virulence factors), and other intestinal components, leading to abnormal immune-inflammatory responses mediated by molecular mimicry and dysregulated T-cell reactions (Yang et al., 2024). A study has shown that IFN-γ expression is increased in mice with tauopathy, and IFN-γ can promote the inflammatory response of microglia, enhancing their antigen presentation and inflammatory functions (Chen et al., 2023). A study has found that Porphyromonas gingivalis (Pg) originating from the oral cavity can induce the proliferation of astrocytes and microglia in the brain. Additionally, the proportion of CD4+IFN-γ+ T lymphocytes and CD8+IFN-γ+ T lymphocytes in the blood and spleen of Pg-treated mice was found to increase (Chi et al., 2021). Peripheral Th1 (CD4^+^ IFN-γ+) cells are associated with M1 microglia activation, suggesting that Pg may affect the brain by inducing glial cell proliferation and promoting the production of pro-inflammatory cytokines through immune cells (Wang et al., 2019). The indirect effects of gut microbiota and its derivatives on microglia are summarized in Figure 2.

### 3.2 Gut microbiota and its derivatives—endocrine system—microglia

#### 3.2.1 Outer membrane vesicles of gut microbiota

Outer membrane vesicles (OMVs) of the gut microbiota are independent vesicles produced by most Gram-negative bacilli and some Gram-positive bacteria. These vesicles can be secreted into the environment distant from the bacterial cells (Juodeikis and Carding, 2022). After entering the systemic circulation, OMVs can affect the central nervous system through various pathways. They can activate innate immune cells in the brain and induce the release of neuroinflammatory factors (Gong et al., 2022). They can also damage or increase the permeability of the blood–brain barrier. Moreover, they may directly interact with neurons, affecting neural activity (Han et al., 2019). On the one hand, OMVs carry lipopolysaccharides, phospholipids, peptidoglycan, cell wall components, proteins, nucleic acids, ion metabolites, and signaling molecules, serving as carriers that play significant roles in biofilm formation, inter-bacterial signaling, antibiotic resistance, regulation of host immune responses, and evasion of host defense systems in physiological and pathological processes (Sartorio et al., 2021). On the other hand, pathogenic gut microbiota (such as *Enterobacteriaceae*, *Shigella*, *Lactobacillus pentosus*, and *Salmonella*) secrete OMVs carrying active virulence factors, which are transported to host cells (Dhital et al., 2021). Adhesins, toxins, and immune regulatory substances within OMVs play a direct role in bacterial adhesion and penetration, leading to cytotoxic effects, and they also regulate the host’s immune response. Through the interaction between the host and the pathogen, OMVs play a crucial role as an important contributor to bacterial virulence. A study found that mice treated with OMVs from *Lactobacillus pentosus* (Lpo) showed a significant decline in cognitive function and hyperphosphorylation of Tau protein compared with the healthy control group, which may be related to the activation of microglia and the production of pro-inflammatory cytokines (IL-1β, IL-6, and TNF-α), thereby exacerbating neuroinflammation (Shao et al., 2024). Another study revealed that OMVs derived from *Helicobacter pylori* (Hpo) can cross biological barriers to reach the brain, where they are taken up by astrocytes, activate glial cells, and lead to neuronal dysfunction, thereby exacerbating Aβ pathology and cognitive decline (Xie et al., 2023).

#### 3.2.2 Neurotransmitters produced by the gut microbiota and its derivatives

The gut microbiota can produce neurotransmitters such as gamma-aminobutyric acid (GABA), noradrenaline (NA), and dopamine (DA), which transmit signals to the brain (Qu et al., 2024; González-Arancibia et al., 2019; Dicks, 2022). GABA is one of the gut microbiota-derived metabolites, with lactic acid bacteria and *Bacteroides* being the main gut bacteria that produce GABA (Bhat et al., 2010; Conn et al., 2024). These bacteria take up glutamate through specific transporters and generate GABA via the L-glutamate decarboxylation reaction within the cell. GABA can cross the blood–brain barrier through simple diffusion, transmembrane transport, or carrier-mediated transport (Takanaga et al., 2001; Al-Sarraf, 2002; Shyamaladevi et al., 2002). Hannah et al. believed that GABA signaling is associated with the activation of microglia and the uptake of Aβ (Iaccarino et al., 2016). Further studies have demonstrated that GABA activates microglia via the NLRP3 inflammasome and NF-κB signaling pathways, leading to significant increases in the mRNA and protein levels of TNF-α, IL-6, and IL-1β in BV2 microglia (Lang et al., 2020). NA is found in the biomass of gut microbiota, with *Escherichia coli*, *Bacillus* amyloliquefaciens, Paenibacillus, *Proteus*, and *Serratia marcescens* being capable of producing NA (Tsavkelova et al., 2000). Moreover, NA can modulate the activation of microglia through β-adrenergic receptors (β-ARs) (Sugama et al., 2019). 5-hydroxytryptamine (5-HT), also known as serotonin, is a neurotransmitter, particularly playing a key role in the gut–brain axis. The gut microbiota influences systemic immune function by regulating the production of 5-HT by enterochromaffin cells in the gut (Correale et al., 2022). Studies have shown that 5-HT regulates neuroinflammation by activating the 5-HT2AR/cAMP/PKA/CREB/Sirt1 and NF-κB pathways, controlling the transcription of TLR2 and TLR4 in microglia stimulated by phagocytosis, thereby affecting neuroinflammation (Lu et al., 2021; Wang et al., 2020; Zhu et al., 2022). In addition, 5-HT can bind to its receptors on microglia, triggering the release of exosomes containing cytokines, providing an alternative mechanism for regulating neuroinflammation caused by the gut (Glebov et al., 2015). The synthesis, metabolism, or transport of 5-HT is crucial in inflammatory responses and may provide new avenues for alleviating neuroinflammation in AD.

### 3.3 Gut microbiota and its derivatives—vagus nerve—microglia

The vagus nerve (VN), as a key component of the autonomic nervous system, contains 80% afferent fibers and 20% efferent fibers, and it intricately traverses the gastrointestinal tract, serving as a critical neural pathway (Bonaz et al., 2021). Gut endocrine cells directly interact with the afferent fibers of the vagus nerve, transmitting information to the central autonomic network for analysis and integration, including the paraventricular nucleus, locus coeruleus, hypothalamus, and the limbic system, which encompasses the thalamus, amygdala, and hippocampus (Schroeder and Bäckhed, 2016). Despite multiple mechanisms, the VN pathway may be the fastest and most direct route of interaction between gut microbiota and the brain (Han et al., 2022). Microbiota indirectly activate the afferent fibers of the vagus nerve through metabolites or other harmful products (such as amyloid proteins and LPS), thereby regulating behaviors such as learning and memory (Décarie-Spain et al., 2024). The connection between gut microbiota and the vagus nerve appears to regulate the state of microglia and the level of inflammation in the central nervous system (Dinan and Cryan, 2017). A study observed that stimulating the vagus nerve can increase the levels of GABA in cerebrospinal fluid and different brain regions. Reactive microglia surrounding amyloid-beta plaques produce high levels of GABA, leading to impaired synaptic plasticity, learning, and memory in AD mice. It is believed that GABA may affect the activation state of microglia through signaling via the vagus nerve (Liang et al., 2024).

## 4 Treatment of AD through gut microbiota regulation

### 4.1 Dietary management

Although the exact etiology of AD remains unclear, it has been recognized that dietary patterns may play a role in the pathological process of AD (Ellouze et al., 2023). In a randomized, double-blind study, it was demonstrated that the modified Mediterranean-ketogenic diet (MMKD) can increase beneficial bacteria (such as Akkermansia and Christensenellaceae), reduce harmful bacteria (such as Enterobacteriaceae and Erysipelotriachaceae), and regulate SCFA levels (increase propionic and butyric acid levels) to improve cognitive function in patients with mild cognitive impairment (MCI) (Nagpal et al., 2019).

### 4.2 Probiotics

In a systematic analysis based on animal and clinical trials, it has been shown that the intake of probiotics has a positive impact on AD, improving memory and cognitive function. Among these, 90% of the studies were based on Bifidobacterium and *Lactobacillus*, while only 10% focused on *Streptococcus* and *Clostridium* species. The most widely used AD preparations are *Bifidobacterium infantis*, *Bifidobacterium longum*, *Lactobacillus acidophilus*, *Lactobacillus plantarum*, and *Lactobacillus casei*, as single- or multi-strain preparations for animal models (Naomi et al., 2021). Although there is evidence supporting the therapeutic potential of probiotics, there are still some risks, such as the potential to induce serotonin syndrome, which requires cautious use in individuals with depression or at risk of AD. Therefore, more research is needed in the future to develop an effective and safe probiotic formulation for the prevention or treatment of AD (Guo et al., 2021).

### 4.3 Fecal microbiota transplantation

Fecal microbiota transplantation (FMT) is a method of transplanting the donor’s gut microbiota into the recipient to restore the gut microbiome. A case report showed that a male patient with recurrent Clostridioides difficile infection (CDI) received an FMT from his wife. After the transplant, the patient’s CDI symptoms resolved, and a significant improvement in his AD symptoms was observed (Hazan, 2020). In another animal study, FMT was performed from non-transgenic WT mice to male APPswe/PS1dE9 transgenic (Tg) mice. It was found that FMT treatment reduced the phosphorylation levels of tau protein and Aβ deposition in the brain, increased synaptic plasticity in Tg mice, suppressed neuroinflammation, reversed changes in the gut microbiota, and increased the levels of butyrate, thereby improving cognitive deficits in Tg mice (Sun J. et al., 2019). Overall, FMT is a safe and effective treatment method, but there are still minor adverse events, such as diarrhea, constipation, abdominal discomfort, and low fever, along with serious side effects, such as high fever, infection, sepsis, transmission of intestinal pathogens, perforation, and bleeding. Therefore, more large-scale clinical trials are needed to further study FMT (Nandwana et al., 2021).

### 4.4 Traditional Chinese medicine

In the past, the mechanism of acupuncture treatment for AD was mainly focused on local changes in the brain. Research in the past decade has shown that acupuncture can regulate the types and structure of gut microbiota, repair the intestinal barrier and blood–brain barrier, prevent inflammatory cytokines from the intestine from entering the blood and brain, and ultimately improve cognitive impairment in AD. Acupuncture at GV20, GV29, ST36, LI4, BL13, BL20, BL23, and SP6 can regulate the composition and quantity of gut microbiota. Acupuncture at GV20, GV29, and ST36 also helps restore the function of the intestinal barrier and blood–brain barrier, reducing inflammatory factors (LPS, TNF-α, and IL-1β) in the circulating blood and brain. Notably, the effectiveness of acupuncture in treating AD is related to the choice of acupuncture points, the form of acupuncture, and the frequency of treatment. Some specific acupuncture methods, such as Sanjiao acupuncture and “Smelling Three Needles” therapy, have also provided new perspectives on the treatment of Alzheimer’s disease (Li et al., 2023). Traditional Chinese medicine (TCM) has a rich theoretical background and clinical experience in the treatment of AD, and it is effective in improving daily living abilities and cognitive functions, alleviating mental symptoms, and delaying disease progression, with the advantages of minimal adverse reactions and high effectiveness (Sun L. et al., 2019). Commonly used TCM compound formulas for treatment include kidney-tonifying and essence-filling formulas such as Liuwei Dihuang Wan, Dihuang Yinzi, and Dabu Yuan Jian; phlegm-removing and orifice-opening formulas such as Kaixin San and Di Tan Tang; blood-activating and stasis-removing formulas such as Buyang Huanwu Tang and Danggui Shaoyao San; heat-clearing and detoxifying formulas such as Huanglian Jiedu Tang; and single-herb treatments such as cooked Rehmannia, dogwood water extract, cinnamon extract, and Artemisia annua water extract. Active components of TCM used in treatment include flavonoids, phenols, quinones, phenylpropanoids, alkaloids, sugars and glycosides, terpenoids, and volatile oils (Cao et al., 2024).

## 5 Summary and outlook

In recent years, an increasing number of studies have shown that increased intestinal permeability or intestinal leakage is associated with AD. Resident immune cells in the central nervous system—microglia—are involved in the development of neuroinflammation. Additionally, due to the role of the microbiota in the maturation and function of microglia, they are mediated by the gut microbiota. Although these articles have determined, based on existing research, that the gut microbiota and its derivatives can regulate microglia to affect AD, these studies may only show us the tip of the iceberg, and there are still many problems that have not been addressed. For example, how does the microbiota promote intestinal permeability, and can this process be reversed (perhaps through fecal transplantation from young mice or probiotics)? What other functions of microglia are altered by the gut microbiota and its derivatives, in addition to activation and inhibition? What are the exact mechanisms through which the gut microbiota and its derivatives influence microglial phenotypes and functional changes? Are there other intestinal-derived metabolites that exert synergistic or antagonistic effects on microglia? Understanding how changes in the gut microbiota and its derivatives affect immune responses in the brain may provide new therapeutic approaches for preventing and treating AD. Therefore, this study aims to elucidate the complex interactions between the gut microbiota, its derivatives, and microglia. Researchers aim to develop innovative approaches targeting the gut–brain axis to improve the prognosis of AD. However, to fully understand the potential of microbiota-based interventions in AD, further efforts are still needed, including the development of non-invasive and *in vivo* monitoring technologies for the composition of the gut microbiota and the function of microglia, along with large-scale clinical trials, to realize therapeutic strategies targeting the microbiota or microglia and benefit patients.

## References

[B1] Abdelhamid M. Zhou C. Ohno K. Kuhara T. Taslima F. Abdullah M. (2022a). Bifidobacterium breve prevents memory impairment through the reduction of both amyloid-β production and microglia activation in app knock-in mouse. J. Alzheimers Dis. 85, 1555-1571. 10.3233/JAD-215025 34958017 PMC8925106

[B2] Abdelhamid M. Zhou C. Jung C. G. Michikawa M. (2022b). Probiotic Bifidobacterium breve MCC1274 mitigates Alzheimer’s disease-related pathologies in wild-type mice. Nutrients 14 (12), 2543. 10.3390/nu14122543 35745273 PMC9231139

[B3] Abraham D. Feher J. Scuderi G. L. Szabo D. Dobolyi A. Cservenak M. (2019). Exercise and probiotics attenuate the development of Alzheimer's disease in transgenic mice: role of microbiome. Exp. Gerontol. 115, 122–131. 10.1016/j.exger.2018.12.005 30529024

[B4] Agus A. Planchais J. Sokol H. (2018). Gut microbiota regulation of tryptophan metabolism in health and disease. Cell host and Microbe 23 (6), 716–724. 10.1016/j.chom.2018.05.003 29902437

[B5] Ahn S. Jin T. E. Chang D. H. Rhee M. S. Kim H. J. Lee S. J. (2016). Agathobaculum butyriciproducens gen. nov. sp. nov., a strict anaerobic, butyrate-producing gut bacterium isolated from human faeces and reclassification of Eubacterium desmolans as Agathobaculum desmolans comb. nov. Int. J. Syst. Evol. Microbiol. 66 (9), 3656–3661. 10.1099/ijsem.0.001195 27334534

[B6] Al-Sarraf H. (2002). Transport of 14C-γ-aminobutyric acid into brain, cerebrospinal fluid and choroid plexus in neonatal and adult rats. Dev. Brain Res. 139 (2), 121–129. 10.1016/s0165-3806(02)00537-0 12480126

[B7] Bhat R. Axtell R. Mitra A. Miranda M. Lock C. Tsien R. W. (2010). Inhibitory role for GABA in autoimmune inflammation. Proc. Natl. Acad. Sci. 107 (6), 2580–2585. 10.1073/pnas.0915139107 20133656 PMC2823917

[B8] Bonaz B. Sinniger V. Pellissier S. (2021). Therapeutic potential of vagus nerve stimulation for inflammatory bowel diseases. Front. Neurosci. 15, 650971. 10.3389/fnins.2021.650971 33828455 PMC8019822

[B9] Borucki D. M. Rothhammer V. J. Quintana F. J. Takenaka M. C. Chao C. C. Ardura-Fabregat A. (2018). Microglial control of astrocytes in response to microbial metabolites. Nature 557, 724–728. 10.1038/s41586-018-0119-x 29769726 PMC6422159

[B10] Brown G. C. Heneka M. T. (2024). The endotoxin hypothesis of Alzheimer’s disease. Mol. Neurodegener. 19 (1), 30. 10.1186/s13024-024-00722-y 38561809 PMC10983749

[B11] Brunt V. E. LaRocca T. J. Bazzoni A. E. Sapinsley Z. J. Miyamoto-Ditmon J. Gioscia-Ryan R. A. (2021). The gut microbiome–derived metabolite trimethylamine N-oxide modulates neuroinflammation and cognitive function with aging. GeroScience 43 (1), 377–394. 10.1007/s11357-020-00257-2 32862276 PMC8050157

[B12] Cao S. Chen Z. Qin J. (2024). Research progress of traditional Chinese medicine and its effective ingredients in the treatment of Alzheimer's disease. Chin. J. Exp. Formulae 30 (10), 258–268. 10.13422/j.cnki.syfjx.20232437

[B13] Chen C. Liao J. Xia Y. Liu X. Jones R. Haran J. (2022). Gut microbiota regulate Alzheimer’s disease pathologies and cognitive disorders via PUFA-associated neuroinflammation. Gut 71 (11), 2233–2252. 10.1136/gutjnl-2021-326269 35017199 PMC10720732

[B14] Chen H. H. Meng L. Shen L. (2022). Multiple roles of short-chain fatty acids in Alzheimer disease. Nutrition 93, 111499. 10.1016/j.nut.2021.111499 34735921

[B15] Chen X. Firulyova M. Manis M. Herz J. Smirnov I. Aladyeva E. (2023). Microglia-mediated T cell infiltration drives neurodegeneration in tauopathy. Nature 615 (7953), 668–677. 10.1038/s41586-023-05788-0 36890231 PMC10258627

[B16] Chen S. Cao Z. Nandi A. Counts N. Jiao L. Prettner K. (2024). The global macroeconomic burden of Alzheimer's disease and other dementias: estimates and projections for 152 countries or territories. Lancet Glob. Health 12 (9), e1534–e1543. 10.1016/S2214-109X(24)00264-X 39151988

[B17] Chi L. Cheng X. Lin L. Yang T. Sun J. Feng Y. (2021). Porphyromonas gingivalis-induced cognitive impairment is associated with gut dysbiosis, neuroinflammation, and glymphatic dysfunction. Front. Cell. Infect. Microbiol. 11, 755925. 10.3389/fcimb.2021.755925 34926316 PMC8672439

[B18] Chilton P. M. Ghare S. S. Charpentier B. T. Myers S. A. Rao A. V. Petrosino J. F. (2024). Age-associated temporal decline in butyrate-producing bacteria plays a key pathogenic role in the onset and progression of neuropathology and memory deficits in 3× Tg-AD mice. Gut Microbes 16 (1), 2389319. 10.1080/19490976.2024.2389319 39182227 PMC11346541

[B19] Conn K. A. Borsom E. M. Cope E. K. (2024). Implications of microbe-derived ɣ-aminobutyric acid (GABA) in gut and brain barrier integrity and GABAergic signaling in Alzheimer’s disease. Gut Microbes 16 (1), 2371950. 10.1080/19490976.2024.2371950 39008552 PMC11253888

[B20] Correale J. Hohlfeld R. Baranzini S. E. (2022). The role of the gut microbiota in multiple sclerosis. Nat. Rev. Neurol. 18 (9), 544–558. 10.1038/s41582-022-00697-8 35931825

[B21] Dammer E. B. Afshar S. Bian S. (2025). “Plasma proteomic associations with alzheimer’s disease endophenotypes,” in Alzheimer's Association International Conference (ALZ). 10.1038/s43587-025-00965-4 PMC12532695 40931114

[B22] Décarie-Spain L. Hayes A. M. R. Lauer L. T. Kanoski S. E. (2024). The gut-brain axis and cognitive control: a role for the vagus nerve. Seminars Cell and Dev. Biol. 156, 201–209. 10.1016/j.semcdb.2023.02.004 36803834 PMC10427741

[B23] Dhital S. Deo P. Stuart I. Naderer T. (2021). Bacterial outer membrane vesicles and host cell death signaling. Trends Microbiol. 29 (12), 1106–1116. 10.1016/j.tim.2021.04.003 34001418

[B24] Di Salvo C. D'Antongiovanni V. Benvenuti L. d'Amati A. Ippolito C. Segnani C. (2024). Lactiplantibacillus plantarum HEAL9 attenuates cognitive impairment and progression of Alzheimer's disease and related bowel symptoms in SAMP8 mice by modulating microbiota-gut-inflammasome-brain axis. Food and Funct. 15 (20), 10323–10338. 10.1039/d4fo02075h 39302233

[B25] Dicks L. M. T. (2022). Gut bacteria and neurotransmitters. Microorganisms 10 (9), 1838. 10.3390/microorganisms10091838 36144440 PMC9504309

[B26] Dinan T. G. Cryan J. F. (2017). Gut-brain axis in 2016: brain-gut-microbiota axis - mood, metabolism and behaviour. Nat. Rev. Gastroenterology and Hepatology 14 (2), 69–70. 10.1038/nrgastro.2016.200 28053341

[B27] Dong F. Perdew G. H. (2020). The aryl hydrocarbon receptor as a mediator of host-microbiota interplay. Gut Microbes 12 (1), 1859812. 10.1080/19490976.2020.1859812 33382356 PMC7781536

[B28] Eid F. Boushehri M. Boucher C. Rajkanth N. Sa A. F. Alhoutan T. (2025). Chronic lipopolysaccharide exposure causes AD‐like pathology in Male mice with intact blood–brain barrier. FASEB J. 39 (9), e70601. 10.1096/fj.202403117RR 40317532

[B29] Ellouze I. Sheffler J. Nagpal R. Arjmandi B. (2023). Dietary patterns and Alzheimer’s disease: an updated review linking nutrition to neuroscience. Nutrients 15, 3204. 10.3390/nu15143204 37513622 PMC10384681

[B30] Erny D. Dokalis N. Mezö C. Castoldi A. Mossad O. Staszewski O. (2021). Microbiota-derived acetate enables the metabolic fitness of the brain innate immune system during health and disease. Cell Metab. 33 (11), 2260–2276. 10.1016/j.cmet.2021.10.010 34731656

[B31] Fathi P. Wu S. (2016). Suppl-1, M3: isolation, detection, and characterization of enterotoxigenic bacteroides fragilis in clinical samples. Open Microbiol. J. 10, 57–63. 10.2174/1874285801610010057 27335618 PMC4899533

[B32] Ferreiro A. L. Choi J. H. Ryou J. Newcomer E. P. Thompson R. Bollinger R. M. (2023). Gut microbiome composition may be an indicator of preclinical Alzheimer’s disease. Sci. Transl. Med. 15 (700), eabo2984. 10.1126/scitranslmed.abo2984 37315112 PMC10680783

[B33] Gao J. Xu K. Liu H. Liu G. Bai M. Peng C. (2018). Impact of the gut microbiota on intestinal immunity mediated by tryptophan metabolism. Front. Cell. Infect. Microbiol. 8, 13. 10.3389/fcimb.2018.00013 29468141 PMC5808205

[B34] Gaugler J. James B. Johnson T. (2019). 2019 Alzheimer's disease facts and figures. Alzheimers and Dementia 15 (3), 321–387. 10.1016/j.jalz.2019.01.010

[B35] Giil L. M. Midttun Ø. Refsum H. Ulvik A. Advani R. Smith A. D. (2017). Kynurenine pathway metabolites in Alzheimer’s disease. J. Alzheimer's Dis. 60 (2), 495–504. 10.3233/JAD-170485 28869479

[B36] Glebov K. Löchner M. Jabs R. Lau T. Merkel O. Schloss P. (2015). Serotonin stimulates secretion of exosomes from microglia cells. Glia 63 (4), 626–634. 10.1002/glia.22772 25451814

[B37] Go J. Chang D. H. Ryu Y. K. Park H. Y. Lee I. B. Noh J. R. (2021). Human gut microbiota Agathobaculum butyriciproducens improves cognitive impairment in LPS-induced and APP/PS1 mouse models of Alzheimer's disease. Nutr. Res. 86, 96–108. 10.1016/j.nutres.2020.12.010 33551257

[B38] Gold A. Kaye S. Gao J. Zhu J. (2024). Propionate decreases microglial activation but impairs phagocytic capacity in response to aggregated fibrillar amyloid beta protein. ACS Chem. Neurosci. 15 (21), 4010–4020. 10.1021/acschemneuro.4c00370 39394077 PMC13135686

[B39] Gong T. Chen Q. Mao H. Zhang Y. Ren H. Xu M. (2022). Outer membrane vesicles of Porphyromonas gingivalis trigger NLRP3 inflammasome and induce neuroinflammation, tau phosphorylation, and memory dysfunction in mice. Front. Cell. Infect. Microbiol. 12, 925435. 10.3389/fcimb.2022.925435 36017373 PMC9397999

[B40] González-Arancibia C. Urrutia-Piñones J. Illanes-González J. Martinez-Pinto J. Sotomayor-Zárate R. Julio-Pieper M. (2019). Do your gut microbes affect your brain dopamine? Psychopharmacology 236, 1611–1622. 10.1007/s00213-019-05265-5 31098656

[B41] Guerreiro R. Wojtas A. Bras J. Carrasquillo M. Rogaeva E. Majounie E. (2013). TREM2 variants in Alzheimer's disease. N. Engl. J. Med. 368 (2), 117–127. 10.1056/NEJMoa1211851 23150934 PMC3631573

[B42] Guo L. Xu J. Du Y. Wu W. Nie W. Zhang D. (2021). Effects of gut microbiota and probiotics on Alzheimer’s disease. Transl. Neurosci. 12 (1), 573–580. 10.1515/tnsci-2020-0203 35070441 PMC8713066

[B43] Han E. C. Choi S. Y. Lee Y. Park J. W. Hong S. H. Lee H. J. (2019). Extracellular RNAs in periodontopathogenic outer membrane vesicles promote TNF-α production in human macrophages and cross the blood–brain barrier in mice. FASEB J. 33 (12), 13412–13422. 10.1096/fj.201901575R 31545910 PMC6894046

[B44] Han Y. Wang B. Gao H. He C. Hua R. Liang C. (2022). Vagus nerve and underlying impact on the gut microbiota-brain axis in behavior and neurodegenerative diseases. J. Inflamm. Res. 15, 6213–6230. 10.2147/JIR.S384949 36386584 PMC9656367

[B45] Hazan S. (2020). Rapid improvement in Alzheimer’s disease symptoms following fecal microbiota transplantation: a case report. J. Int. Med. Res. 48 (6), 0300060520925930. 10.1177/0300060520925930 32600151 PMC7328362

[B46] Hunter S. Walsh S. Brayne C. (2025). Key questions for the future of amyloid research in dementia: a framework for integrating complex datasets. Mol. Psychiatry 30, 5001–5010. 10.1038/s41380-025-03156-0 40847003 PMC12436173

[B47] Iaccarino H. F. Singer A. C. Martorell A. J. Rudenko A. Gao F. Gillingham T. Z. (2016). Gamma frequency entrainment attenuates amyloid load and modifies microglia. Nature 540 (7632), 230–235. 10.1038/nature20587 27929004 PMC5656389

[B48] Jia W. Rajani C. Kaddurah‐Daouk R. Li H. (2020). Expert insights: the potential role of the gut microbiome‐bile acid‐brain axis in the development and progression of Alzheimer's disease and hepatic encephalopathy. Med. Res. Rev. 40 (4), 1496–1507. 10.1002/med.21653 31808182

[B49] Jia L. Ke Y. Zhao S. Liu J. Luo X. Cao J. (2025). Metagenomic analysis characterizes stage-specific gut microbiota in Alzheimer’s disease. Mol. Psychiatry 30, 3951–3962. 10.1038/s41380-025-02973-7 40164697

[B50] Jiang Y. Li K. Li X. Xu L. Yang Z. (2021). Sodium butyrate ameliorates the impairment of synaptic plasticity by inhibiting the neuroinflammation in 5XFAD mice. Chemico-biological Interact. 341, 109452. 10.1016/j.cbi.2021.109452 33785315

[B51] Jonsson T. Stefansson H. Steinberg S. Jonsdottir I. Jonsson P. V. Snaedal J. (2013). Variant of TREM2 associated with the risk of Alzheimer's disease. N. Engl. J. Med. 368 (2), 107–116. 10.1056/NEJMoa1211103 23150908 PMC3677583

[B52] Juodeikis R. Carding S. R. (2022). Outer membrane vesicles: biogenesis, functions, and issues. Microbiol. Mol. Biol. Rev. 86 (4), e0003222–e0003222. 10.1128/mmbr.00032-22 36154136 PMC9881588

[B53] Kang J. W. Khatib L. A. Heston M. B. Dilmore A. H. Labus J. S. Deming Y. (2025). Gut microbiome compositional and functional features associate with Alzheimer's disease pathology. Alzheimer's and Dementia 21 (7), e70417. 10.1002/alz.70417 40604345 PMC12221809

[B54] Keane L. Clarke G. Cryan J. F. (2025). A role for microglia in mediating the microbiota–gut–brain axis. Nat. Rev. Immunol. 25, 847–861. 10.1038/s41577-025-01188-9 40506470

[B55] Kesika P. Suganthy N. Sivamaruthi B. S. Chaiyasut C. (2021). Role of gut-brain axis, gut microbial composition, and probiotic intervention in Alzheimer's disease. Life Sci. 264, 118627. 10.1016/j.lfs.2020.118627 33169684

[B56] Khoshnevisan K. Chehrehgosha M. Conant M. Meftah A. M. Baharifar H. Ejtahed H. S. (2022). Interactive relationship between Trp metabolites and gut microbiota: the impact on human pathology of disease. J. Appl. Microbiol. 132 (6), 4186–4207. 10.1111/jam.15533 35304801

[B57] Kurita N. Yamashiro K. Kuroki T. Tanaka R. Urabe T. Ueno Y. (2020). Metabolic endotoxemia promotes neuroinflammation after focal cerebral ischemia. J. Cereb. Blood Flow and Metabolism 40 (12), 2505–2520. 10.1177/0271678X19899577 31910709 PMC7820690

[B58] Lang L. Xu B. Yuan J. Li S. Lian S. Chen Y. (2020). GABA-mediated activated microglia induce neuroinflammation in the hippocampus of mice following cold exposure through the NLRP3 inflammasome and NF-κB signaling pathways. Int. Immunopharmacol. 89, 106908. 10.1016/j.intimp.2020.106908 33166810

[B59] Lee H. J. Hwang Y. H. Kim D. H. (2018). Lactobacillus plantarum C29‐fermented soybean (DW2009) alleviates memory impairment in 5XFAD transgenic mice by regulating microglia activation and gut microbiota composition. Mol. Nutr. and Food Res. 62 (20), 1800359. 10.1002/mnfr.201800359 30152045

[B60] Lei W. Cheng Y. Liu X. Gao J. Zhu Z. Ding W. (2025). Gut Microbiota-driven neuroinflammation in Alzheimer's disease: from mechanisms to therapeutic opportunities. Front. Immunol. 16, 1582119. 10.3389/fimmu.2025.1582119 40642089 PMC12241022

[B61] Li N. Kang X. Zhao T. (2023). The effectiveness and mechanisms of acupuncture in treating Alzheimer's disease.

[B62] Liang J. Wang Y. Liu B. Dong X. Cai W. Zhang N. (2024). Deciphering the intricate linkage between the gut microbiota and Alzheimer's disease: elucidating mechanistic pathways promising therapeutic strategies. CNS Neurosci. and Ther. 30 (4), e14704. 10.1111/cns.14704 38584341 PMC10999574

[B63] Ling Z. Zhu M. Yan X. Cheng Y. Shao L. Liu X. (2021). Structural and functional dysbiosis of fecal microbiota in Chinese patients with Alzheimer's disease. Front. Cell Dev. Biol. 8, 634069. 10.3389/fcell.2020.634069 33614635 PMC7889981

[B64] Liu J. Li H. Gong T. Chen W. Mao S. Kong Y. (2020). Anti-neuroinflammatory effect of short-chain fatty acid acetate against Alzheimer’s disease via upregulating GPR41 and inhibiting ERK/JNK/NF-κB. J. Agric. Food Chem. 68 (27), 7152–7161. 10.1021/acs.jafc.0c02807 32583667

[B65] Liu L. Tong F. Li H. Bin Y. Ding P. Peng L. (2023). Maturation, morphology, and function: the decisive role of intestinal flora on microglia: a review. J. Integr. Neurosci. 22 (3), 70. 10.31083/j.jin2203070 37258438

[B66] Lopez J. A. S. González H. M. Léger G. C. (2019). Alzheimer's disease. Handb. Clin. Neurology 167, 231–255. 10.1016/B978-0-12-804766-8.00013-3 31753135

[B67] Lu J. Zhang C. Lv J. Zhu X. Jiang X. Lu W. (2021). Antiallergic drug desloratadine as a selective antagonist of 5HT2A receptor ameliorates pathology of Alzheimer's disease model mice by improving microglial dysfunction. Aging Cell 20 (1), e13286. 10.1111/acel.13286 33369003 PMC7811850

[B68] Lukiw W. J. (2016). Bacteroides fragilis lipopolysaccharide and inflammatory signaling in Alzheimer’s disease. Front. Microbiol. 7, 1544. 10.3389/fmicb.2016.01544 27725817 PMC5035737

[B69] Lv X. Zhan L. Ye T. Xie H. Chen Z. Lin Y. (2024). Gut commensal Agathobacter rectalis alleviates microglia-mediated neuroinflammation against pathogenesis of Alzheimer disease. iScience 27 (11), 111116. 10.1016/j.isci.2024.111116 39498309 PMC11532950

[B70] Lyu J. Xie D. Bhatia T. N. Leak R. K. Hu X. Jiang X. (2021). Microglial/Macrophage polarization and function in brain injury and repair after stroke. CNS Neurosci. and Ther. 27 (5), 515–527. 10.1111/cns.13620 33650313 PMC8025652

[B71] Ma N. He T. Johnston L. J. Ma X. (2020). Host–microbiome interactions: the aryl hydrocarbon receptor as a critical node in tryptophan metabolites to brain signaling. Gut Microbes 11 (5), 1203–1219. 10.1080/19490976.2020.1758008 32401136 PMC7524279

[B72] Ma J. Li M. Bao Y. Huang W. He X. Hong Y. (2024). Gut microbiota-brain bile acid axis orchestrates aging-related neuroinflammation and behavior impairment in mice. Pharmacol. Res. 208, 107361. 10.1016/j.phrs.2024.107361 39159729

[B73] MahmoudianDehkordi S. Arnold M. Nho K. Ahmad S. Jia W. Xie G. (2019). Altered bile acid profile associates with cognitive impairment in Alzheimer's disease—an emerging role for gut microbiome. Alzheimer's and Dementia 15 (1), 76–92. 10.1016/j.jalz.2018.07.217 30337151 PMC6487485

[B74] Malaguarnera M. Bella R. Alagona G. Ferri R. Carnemolla A. Pennisi G. (2004). Helicobacter pylori and Alzheimer's disease: a possible link. Eur. J. Intern. Med. 15 (6), 381–386. 10.1016/j.ejim.2004.05.008 15522573

[B75] Mou Y. Du Y. Zhou L. Yue J. Hu X. Liu Y. (2022). Gut microbiota interact with the brain through systemic chronic inflammation: implications on neuroinflammation, neurodegeneration, and aging. Front. Immunol. 13, 796288. 10.3389/fimmu.2022.796288 35464431 PMC9021448

[B76] Nagpal R. Neth B. J. Wang S. Craft S. Yadav H. (2019). Modified Mediterranean-ketogenic diet modulates gut microbiome and short-chain fatty acids in association with Alzheimer's disease markers in subjects with mild cognitive impairment. EBioMedicine 47, 529–542. 10.1016/j.ebiom.2019.08.032 31477562 PMC6796564

[B77] Nandwana V. Debbarma S. Singh R. Jaka S. Kaur G. Rawal E. (2021). Predictors of hospitalization for manic episode in Alzheimer's dementia: inputs from an inpatient case-control study. Cureus 13 (7), e17333. 10.7759/cureus.17333 34567877 PMC8451530

[B78] Naomi R. Embong H. Othman F. Ghazi H. F. Maruthey N. Bahari H. (2021). Probiotics for Alzheimer’s disease: a systematic review. Nutrients 14 (1), 20. 10.3390/nu14010020 35010895 PMC8746506

[B79] Obermeier B. Daneman R. Ransohoff R. M. (2013). Development, maintenance and disruption of the blood-brain barrier. Nat. Med. 19 (12), 1584–1596. 10.1038/nm.3407 24309662 PMC4080800

[B80] Pan S. Zhang Y. Ye T. Kong Y. Cui X. Yuan S. (2024). A high‐tryptophan diet alleviated cognitive impairment and neuroinflammation in APP/PS1 mice through activating aryl hydrocarbon receptor via the regulation of gut microbiota. Mol. Nutr. and Food Res. 68 (2), 2300601. 10.1002/mnfr.202300601 38031265

[B81] Pellegrini C. Antonioli L. Colucci R. Blandizzi C. Fornai M. (2018). Interplay among gut microbiota, intestinal mucosal barrier and enteric neuro-immune system: a common path to neurodegenerative diseases? Acta Neuropathol. 136, 345–361. 10.1007/s00401-018-1856-5 29797112

[B82] Praveenraj S. S. Sonali S. Anand N. Tousif H. A. Vichitra C. Kalyan M. (2022). The role of a gut microbial-derived metabolite, trimethylamine N-oxide (TMAO), in neurological disorders. Mol. Neurobiol. 59 (11), 6684–6700. 10.1007/s12035-022-02990-5 35986843

[B83] Qian X. Hai W. Chen S. Zhang M. Jiang X. Tang H. (2023). Multi-omics data reveals aberrant gut microbiota-host glycerophospholipid metabolism in association with neuroinflammation in APP/PS1 mice. Gut Microbes 15 (2), 2282790. 10.1080/19490976.2023.2282790 37992400 PMC10730179

[B84] Qu S. Yu Z. Zhou Y. Wang S. Jia M. Chen T. (2024). Gut microbiota modulates neurotransmitter and gut-brain signaling. Microbiol. Res. 287, 127858. 10.1016/j.micres.2024.127858 39106786

[B85] Rafii M. S. Aisen P. S. (2025). Amyloid-lowering immunotherapies for Alzheimer disease: current status and future directions. Nat. Rev. Neurol. 21, 490–498. 10.1038/s41582-025-01123-5 40691719

[B86] Rothhammer V. Mascanfroni I. D. Bunse L. Takenaka M. C. Kenison J. E. Mayo L. (2016). Type I interferons and microbial metabolites of tryptophan modulate astrocyte activity and central nervous system inflammation via the aryl hydrocarbon receptor. Nat. Med. 22 (6), 586–597. 10.1038/nm.4106 27158906 PMC4899206

[B87] Sartorio M. G. Pardue E. J. Feldman M. F. Haurat M. F. (2021). Bacterial outer membrane vesicles: from discovery to applications. Annu. Rev. Microbiol. 75 (1), 609–630. 10.1146/annurev-micro-052821-031444 34351789 PMC8500939

[B88] Scheltens P. De Strooper B. Kivipelto M. Holstege H. Chételat G. Teunissen C. E. (2021). Alzheimer's disease. Lancet 397 (10284), 1577–1590. 10.1016/S0140-6736(20)32205-4 33667416 PMC8354300

[B89] Schroeder B. O. Bäckhed F. (2016). Signals from the gut microbiota to distant organs in physiology and disease. Nat. Med. 22 (10), 1079–1089. 10.1038/nm.4185 27711063

[B90] Sender R. Fuchs S. Milo R. (2016). Are we really vastly outnumbered? Revisiting the ratio of bacterial to host cells in humans. Cell 164 (3), 337–340. 10.1016/j.cell.2016.01.013 26824647

[B91] Shao Z. Lu Y. Xing A. He X. Xie H. Hu M. (2024). Effect of outer membrane vesicles of Lactobacillus pentosus on Tau phosphorylation and CDK5-Calpain pathway in mice. Exp. Gerontol. 189, 112400. 10.1016/j.exger.2024.112400 38484904

[B92] Shi F. D. Yong V. W. (2025). Neuroinflammation across neurological diseases. Science 388 (6753), eadx0043. 10.1126/science.adx0043 40536983

[B93] Shyamaladevi N. Jayakumar A. R. Sujatha R. Paul V. Subramanian E. H. (2002). Evidence that nitric oxide production increases γ-amino butyric acid permeability of blood-brain barrier. Brain Res. Bull. 57 (2), 231–236. 10.1016/s0361-9230(01)00755-9 11849830

[B94] Sugama S. Takenouchi T. Hashimoto M. Ohata H. Takenaka Y. Kakinuma Y. (2019). Stress-induced microglial activation occurs through β-adrenergic receptor: noradrenaline as a key neurotransmitter in microglial activation. J. Neuroinflammation 16, 266–16. 10.1186/s12974-019-1632-z 31847911 PMC6916186

[B95] Sun J. Xu J. Ling Y. Wang F. Gong T. Yang C. (2019). Fecal microbiota transplantation alleviated Alzheimer’s disease-like pathogenesis in APP/PS1 transgenic mice. Transl. Psychiatry 9 (1), 189. 10.1038/s41398-019-0525-3 31383855 PMC6683152

[B96] Sun L. Zhan M. He C. (2019). Research progress of traditional Chinese medicine in the treatment of Alzheimer's disease. J. Integr. Chin. West. Med. Cardio-Cerebrovascular Dis. 19 (19), 3323–3328.

[B97] Sun J. J. Xu J. Yang B. Chen K. Kong Y. Fang N. (2020). Effect of Clostridium butyricum against microglia‐mediated neuroinflammation in Alzheimer's disease via regulating gut microbiota and metabolites butyrate. Mol. Nutr. and Food Res. 64 (2), 1900636. 10.1002/mnfr.201900636 31835282

[B98] Sun M. Ma N. He T. Johnston L. J. Ma X. (2020). Tryptophan (Trp) modulates gut homeostasis via aryl hydrocarbon receptor (AhR). Crit. Rev. Food Sci. Nutr. 60 (10), 1760–1768. 10.1080/10408398.2019.1598334 30924357

[B99] Sun J. Zhang Y. Kong Y. Yu Q. Kumaran Satyanarayanan S. (2022). Microbiota-derived metabolite indoles induced aryl hydrocarbon receptor activation and inhibited neuroinflammation in APP/PS1 mice. Brain, Behav. Immun. 106, 76–88. 10.1016/j.bbi.2022.08.003 35961580

[B100] Takanaga H. Ohtsuki S. Hosoya K. I. Terasaki T. (2001). GAT2/BGT-1 as a system responsible for the transport of γ-aminobutyric acid at the mouse blood–brain barrier. J. Cereb. Blood Flow and Metabolism 21 (10), 1232–1239. 10.1097/00004647-200110000-00012 11598501

[B101] Teng Y. Mu J. Xu F. Zhang X. Sriwastva M. K. Liu Q. M. (2022). Gut bacterial isoamylamine promotes age-related cognitive dysfunction by promoting microglial cell death. Cell Host and Microbe 30 (7), 944–960. 10.1016/j.chom.2022.05.005 35654045 PMC9283381

[B102] Tsavkelova E. A. Botvinko I. V. Kudrin V. S. Oleskin A. V. (2000). Detection of neurotransmitter amines in microorganisms with the use of high-performance liquid chromatography. Dokl. Biochem. 372 (1-6), 115–117. 10935181

[B103] Uberti A. F. Callai-Silva N. Grahl M. V. C. Piovesan A. R. Nachtigall E. G. Furini C. R. G. (2022). *Helicobacter pylori* urease: potential contributions to Alzheimer’s disease. Int. J. Mol. Sci. 23 (6), 3091. 10.3390/ijms23063091 35328512 PMC8949269

[B104] Vighi G. Marcucci F. Sensi L. Di Cara G. Frati F. (2008). Allergy and the gastrointestinal system. Clin. and Exp. Immunol. 153 (Suppl. ment_1), 3–6. 10.1111/j.1365-2249.2008.03713.x 18721321 PMC2515351

[B105] Wang X. Sun G. Feng T. Zhang J. Huang X. Wang T. (2019). Sodium oligomannate therapeutically remodels gut microbiota and suppresses gut bacterial amino acids-shaped neuroinflammation to inhibit Alzheimer’s disease progression. Cell Res. 29 (10), 787–803. 10.1038/s41422-019-0216-x 31488882 PMC6796854

[B106] Wang M. Zong H. F. Chang K. W. Yasir Rizvi M. Iffat Neha S. (2020). 5-HT1AR alleviates Aβ-induced cognitive decline and neuroinflammation through crosstalk with NF-κB pathway in mice. Int. Immunopharmacol. 82, 106354. 10.1016/j.intimp.2020.106354 32143008

[B107] Wang P. Mi Y. Yu H. Teng X. Jin S. Xiao L. (2024). Trimethylamine-N-oxide aggravated the sympathetic excitation in D-galactose induced aging rats by down-regulating P2Y12 receptor in microglia. Biomed. and Pharmacother. 174, 116549. 10.1016/j.biopha.2024.116549 38593701

[B108] Wang Z. Zhang L. Qin C. (2025). Alzheimer’s disease pathogenesis: standing at the crossroad of lipid metabolism and immune response. Mol. Neurodegener. 20 (1), 67. 10.1186/s13024-025-00857-6 40468377 PMC12139291

[B109] Wasén C. Beauchamp L. C. Vincentini J. Li S. LeServe D. S. Gauthier C. (2024). Bacteroidota inhibit microglia clearance of amyloid-beta and promote plaque deposition in Alzheimer’s disease mouse models. Nat. Commun. 15 (1), 3872. 10.1038/s41467-024-47683-w 38719797 PMC11078963

[B110] Wenzel T. J. Gates E. J. Ranger A. L. Klegeris A. (2020). Short-chain fatty acids (SCFAs) alone or in combination regulate select immune functions of microglia-like cells. Mol. Cell. Neurosci. 105, 103493. 10.1016/j.mcn.2020.103493 32333962

[B111] Wightman D. P. Jansen I. E. Savage J. E. Shadrin A. A. Bahrami S. Holland D. (2021). A genome-wide association study with 1,126,563 individuals identifies new risk loci for Alzheimer’s disease. Nat. Genet. 53 (9), 1276–1282. 10.1038/s41588-021-00921-z 34493870 PMC10243600

[B112] Wu Q. Li Q. Zhang X. Ntim M. Wu X. Li M. (2020). Treatment with bifidobacteria can suppress Aβ accumulation and neuroinflammation in APP/PS1 mice. PeerJ 8, e10262. 10.7717/peerj.10262 33194428 PMC7602682

[B113] Xia Y. Xiao Y. Wang Z. H. Liu X. Alam A. M. Haran J. P. (2023). Bacteroides Fragilis in the gut microbiomes of Alzheimer’s disease activates microglia and triggers pathogenesis in neuronal C/EBPβ transgenic mice. Nat. Commun. 14 (1), 5471. 10.1038/s41467-023-41283-w 37673907 PMC10482867

[B114] Xie J. Cools L. Van Imschoot G. Van Wonterghem E. Pauwels M. J. Vlaeminck I. (2023). Helicobacter pylori‐derived outer membrane vesicles contribute to Alzheimer's disease pathogenesis via C3‐C3aR signalling. J. Extracell. Vesicles 12 (2), 12306. 10.1002/jev2.12306 36792546 PMC9931688

[B115] Yang J. Liang J. Hu N. He N. Liu B. Liu G. (2024). The gut microbiota modulates neuroinflammation in Alzheimer's disease: elucidating crucial factors and mechanistic underpinnings. CNS Neurosci. and Ther. 30 (10), e70091. 10.1111/cns.70091 39460538 PMC11512114

[B116] Yoo B. B. Mazmanian S. K. (2017). The enteric network: interactions between the immune and nervous systems of the gut. Immunity 46 (6), 910–926. 10.1016/j.immuni.2017.05.011 28636959 PMC5551410

[B117] Zarbock K. R. Han J. H. Singh A. P. Thomas S. P. Bendlin B. B. Denu J. M. (2022). Trimethylamine N-oxide reduces neurite density and plaque intensity in a murine model of Alzheimer’s disease. J. Alzheimer's Dis. 90 (2), 585–597. 10.3233/JAD-220413 36155509 PMC9881463

[B118] Zhang J. Zhang Y. Wang J. Xia Y. Chen L. (2024). Recent advances in Alzheimer’s disease: mechanisms, clinical trials and new drug development strategies. Signal Transduct. Target. Ther. 9 (1), 211. 10.1038/s41392-024-01911-3 39174535 PMC11344989

[B119] Zhang S. Gao Y. Zhao Y. Huang T. Y. Zheng Q. Wang X. (2025). Peripheral and central neuroimmune mechanisms in Alzheimer’s disease pathogenesis. Mol. Neurodegener. 20 (1), 22. 10.1186/s13024-025-00812-5 39985073 PMC11846304

[B120] Zhao Y. Lukiw W. J. (2018). Bacteroidetes neurotoxins and inflammatory neurodegeneration. Mol. Neurobiol. 55, 9100–9107. 10.1007/s12035-018-1015-y 29637444

[B121] Zhu P. Lu T. Wu J. Fan D. Liu B. Zhu X. (2022). Gut microbiota drives macrophage-dependent self-renewal of intestinal stem cells via niche enteric serotonergic neurons. Cell Res. 32 (6), 555–569. 10.1038/s41422-022-00645-7 35379903 PMC9160288

[B122] Zhu G. Zhao J. Wang G. Chen W. (2023). Bifidobacterium breve HNXY26M4 attenuates cognitive deficits and neuroinflammation by regulating the gut–brain axis in APP/PS1 mice. J. Agric. Food Chem. 71 (11), 4646–4655. 10.1021/acs.jafc.3c00652 36888896

